# Descriptive Study on Parents’ Knowledge, Attitudes and Practices on Antibiotic Use and Misuse in Children with Upper Respiratory Tract Infections in Cyprus

**DOI:** 10.3390/ijerph8083246

**Published:** 2011-08-05

**Authors:** Andreas Rousounidis, Vassiliki Papaevangelou, Adamos Hadjipanayis, Sotiria Panagakou, Maria Theodoridou, George Syrogiannopoulos, Christos Hadjichristodoulou

**Affiliations:** 1 Department of Hygiene and Epidemiology, Faculty of Medicine, University of Thessaly, 22 Papakyriazi street, Larissa 41222, Greece; E-Mails: rousounides@cytanet.com.cy (A.R.); geopanagakos@yahoo.gr (S.P.); 2 Second Department of Pediatrics, P&A Kyriakou Children’s Hospital, University of Athens, Athens 10679, Greece; E-Mail: vpapaev@med.uoa.gr; 3 Department of Pediatrics, Larnaca General Hospital, Larnaca, Cyprus; E-Mail: adahadji@cytanet.com.cy; 4 First Department of Pediatrics, Aghia Sophia Children’s Hospital, University of Athens, Athens 10679, Greece; E-Mail: alexandratheo@yahoo.gr; 5 Department of Pediatrics, Faculty of Medicine, University of Thessaly, General University Hospital, Larisa 41222, Greece; E-Mail: syrogian@otenet.gr

**Keywords:** antibiotics, bacterial resistance, KAP study, knowledge, attitudes, practices, antibiotic overuse, antibiotic misuse, questionnaire, parents

## Abstract

Upper respiratory tract infections (URTIs) are common in children and represent a significant cause of antibiotic abuse which contributes to the development of antibiotic resistance. A survey was conducted in Cyprus in 2006 to assess parents’ and pediatricians’ Knowledge, Attitude and Practices (KAP) concerning the role of antibiotics in children with URTIs. A school-based stratified geographic clustering sampling was used and a pre-tested KAP questionnaire was distributed. A different questionnaire was distributed to paediatricians. Demographic factors associated with antibiotic misuse were identified by backward logistic regression analysis. The parental overall response rate was 69.3%. Parents (N = 1,462) follow pediatricians advice and rarely administer antibiotics acquired over the counter. Although a third expects an antibiotic prescription for URTI symptoms, most deny pressuring their doctors. Low parental education was the most important independent risk factor positively related to antibiotic misuse (OR = 2.88, 95%CI 2.02 to 4.12, p < 0.001). Pediatricians (N = 33) denied prescribing antibiotics after parental pressure but admit that parents ask for antibiotics and believe they expect antibiotic prescriptions even when not needed. In conclusion, Cypriotic parents trust their primary care providers. Although it appears that antibiotic misuse is not driven by parental pressure, the pediatricians’ view differs.

## 1. Introduction

The emergence of bacterial strains resistant to antimicrobial agents presents a growing concern worldwide [1]. The relationship between antibiotic use and resistance development is strong and supported by several studies [2,3]. Countries with the highest *per capita* antibiotic consumption have the highest prevalence of resistant pathogens [4,5]. Antibiotics are often used for the treatment of upper respiratory tract infections, including sore throat, common cold and rhinitis, even though viruses cause most of these illnesses [6]. Antibiotics abuse in upper respiratory tract infections (URTIs) in children is an important factor contributing to the development of antibiotic resistance and therefore the judicious use of antibiotics in pediatric clinical practice is crucial [7]. It has been estimated that 20–50% of all antimicrobial use is inappropriate [8]. Factors leading to antimicrobial overuse in children are complex, involving, among other factors, parental knowledge and attitude, physician beliefs as well as constraints of daily practice [8–11].

There is limited data regarding the use of antimicrobials and the prevalence of resistance among respiratory pathogens in Cyprus. Recent published data revealed that overall antibiotic consumption in Cyprus during the years 2006–2008, was higher than other EU countries (32.78 DDD/1,000 inhabitants/day) with about 75% consumption taking place in the private sector. Beta-lactam antibiotics represented 60% of total antibiotic consumption [12]. Although a slight decrease has been observed on beta-lactam antibiotic consumption over the recent years, a worrisome increase in the quinolone consumption has been recorded [12]. Preliminary results from the Antibiotic Resistance Surveillance and Control in the Mediterranean Region (ARMed) project further supports previous sporadic reports suggesting high antibiotic resistance in this region [13].

As no similar literature exists from this country, this study aims to explore the parental knowledge, attitudes and practices (KAP) regarding antibiotic use for children 4–7 years old, in Cyprus. Moreover, to investigate primary care physicians’ view on this subject, paediatricians practicing at the same areas were asked to fill another KAP questionnaire. This survey is the first part of a national intervention program aiming to monitor and reduce antibiotic misuse in children.

## 2. Methods

### 2.1. Participants

The study was carried out in two districts of Cyprus, Larnaca and Limassol, between 1 February and 13 May 2006. These are two mainly urban areas with similar demographic characteristics and roughly 200,000 population each. The protocol was in accordance to the Declaration of Helsinki (1989) of the World Medical Association and was approved by the ethics committee of the Cypriot Ministry of Education which accepted the waiver of consent. A school based stratified geographic clustering sampling was used to select a representative sample of children 4–7 years old. The list of all Cyprus kindergarten and elementary schools was provided by the Ministry of Education and stratification was conducted using the population density of the two districts of Cyprus, Larnaca and Limassol. The schools were randomly selected, using a cross-sectional design, in order to obtain an unbiased sample of the population in the areas studied regarding age, gender and socioeconomic status. In total, 18 kindergarten (10 in Larnaca and eight in Limassol) and 15 elementary schools (seven and eight, respectively) were randomly selected. All parents of children attending the kindergartens and elementary schools were asked to participate. In a second sub-study, we included all pediatricians practicing in these two districts and asked them to complete a specially designed KAP questionnaire addressing antibiotic consumption in children with URTIs.

### 2.2. Procedures

After receiving written permission from the Ministry of Education, the questionnaire was distributed by teachers to all children attending the schools. In order to increase compliance, all teachers where personally informed by the researcher about the study and its importance. They were asked to distribute the questionnaires to their students, collect and mail them back to the researcher. With each questionnaire, a formal note addressing the parents, explaining the importance of the subject and their co-operation was handed out.

### 2.3. Questionnaires

A pre-tested questionnaire previously developed by our team was used in this survey [14]. The KAP-questionnaire, included demographic data and was structured in three main sections which displayed the Knowledge (Section A), Attitudes (Section B) and Practices (Section C) of parents regarding antibiotics use in upper respiratory track infections (URTI) of their children.

Demographic data included age, gender, socioeconomic, educational and health insurance status. Section A included questions regarding parental knowledge concerning antibiotics. They were asked to mark antibiotic names out of ten commonly used medications and to answer questions relevant to antibiotics indications, side effects and their use in viral infections. Section B studied the parental attitudes regarding URTIs, antimicrobial agents’ use and the pediatricians’ role. Parents where asked which symptoms and what duration would lead them to seek medical attention for their children, as well as their expectations regarding antibiotics prescription. Other questions included reasons for antibiotic use without medical advice (over the counter acquisition of antibiotic, use of leftover antibiotic from previous illness, *etc.*) or whether they would seek for a pediatrician who is more lenient with antibiotic administration. Finally, section C looked into parental practices and whether the parent-doctor relation is influenced by the latter’s attitude on antibiotic prescription. Parents were asked whether their pediatrician spends enough time explaining the illness and suggested antibiotic treatment for their child, whether he is influenced by their demand to prescribe antibiotics, as well as whether their pediatrician gives them instructions over the phone (without previous examination) for antibiotic administration to their sick child.

Each question (apart from those included in the demographic data section) was in a format of five possible answers (accepting only one right answer), according to the 5-point Likert scale: 1 = strongly agree, 2 = agree, 3 = uncertain, 4 = disagree and 5 = disagree strongly or 1 = always, 2 = most of the times, 3 = often, 4 = sometimes and 5 = never.

To verify parent’s responses consistency and exclude random completion, three couples of similar questions (where each couple included the same statement expressed in a different way) and three pairs of contradictory questions (where each question included the reverse statement requiring the opposite answer) were entered in the questionnaire’s structure. All these questions were randomly placed in the questionnaire to minimize parents’ awareness. Questionnaires with discordant responses to two or more of these paired questions were removed.

To further analyze demographic factors associated with antibiotic misuse logistic regression analysis was applied. Six groups of questions (G1–G6), best predicting antibiotic misuse were selected as shown in Table 1.

A second questionnaire, distributed to pediatricians, included nameless demographics, data concerning the level of postgraduate studies as well as three sections referring to their Knowledge, Attitudes and Practices regarding antibiotic prescription to children with URTIs. This questionnaire has not been pre-tested, had similar format to the parental one but did not include similar-contradictory questions. To increase information accuracy questionnaires were anonymous and special care was taken during collection to prevent accidental identification.

### 2.4. Statistical Analysis

The data were entered in a database created by using the Epi Info software. The statistical analysis was conducted by using the Epi Info (http://www.cdc.gov/) and SPSS software version 15.0. Parents’ answers were analyzed using the 5-point Likert scale. More specifically, the possible answers “strongly agree” and “agree” were considered as a “positive” answer, while the possible answers “disagree” and “strongly disagree” were considered as a “negative” answer. The possible answer “uncertain” was not taken into consideration. Similarly, the possible answers “always”, “most of the times” were considered as “frequently”, while the possible answers “sometimes” and “never” were considered as “rarely”. The possible answer “often” was not taken into consideration. Univariate analysis was used to identify risk factors associated to injudicious antibiotic use. Chi-square test or Fisher’s exact test were used for qualitative data. Relative Risk (RR) and its 95% Confidence Interval were calculated as well. Statistically significant factors were included in a conditional backward logistic regression model. A p-value < 0.05 was considered statistically significant.

## 3. Results

Overall, 2,155 questionnaires were distributed. The overall response rate was 69.3% (1,494/2,155); and was significantly higher in Larnaca (77.4%, 778/1,005) compared to Limassol area (62.3%, 716/1,150) (p < 0.001). Twenty five questionnaires (1.7%) were excluded from further analysis since parents had contradictory responses to two or more of the six couples of similar-contradictive questions. Furthermore, another seven questionnaires were excluded from further analysis since parents did not respond to more than 50% of the questions. Therefore 1,462 questionnaires were analyzed.

### 3.1. Demographic Data

Parent’s demographics are shown in Table 2. Thirty three pediatricians (33/40 enrolled, 17 pediatricians from Larnaca), returned their completed KAP questionnaires. Most pediatricians were males (22/33), over 50 years old (18/33), general pediatricians without subspecialty (28/33) in private practice (29/33).

### 3.2. Parental KAP

#### 3.2.1. Section A (Knowledge)

Most parents (90%) affirmed that their pediatrician was the main source for information regarding antibiotics, while television (15.3%) and newspaper (11.6%) followed. However, 3.1% of parents declared they had never received any information. Out of ten commonly used medications, most parents (64%) were able to identify 3/5 antibiotic names.

Although the majority of parents acknowledged that antibiotics have side effects (93%), agreed that misuse reduces their efficiency and increases bacteria resistance (90%), that viral URTI’s are self limited (60%) and that fever is not an indication for the administration of antibiotics (87%), many (48.4%) believed that antibiotics may decrease duration of URTI symptoms. Interestingly, most parents (85%) believed that scientists continuously discover new antimicrobial agents against resistant microorganisms and that antibiotics may decrease the incidence of URTI complications (73.5%).

#### 3.2.2. Section B (Attitudes)

Most parents stated that they would seek medical advice for their children, after 2 days of upper respiratory tract infection symptoms. The main symptoms considered important and would drive parents to the doctor’s office included earache (84%) and fever (81%), followed by sore throat (45%), cough (36%) and altered behavior (33%). Many parents (33.5%) acknowledged that they expect from their pediatrician to prescribe an antibiotic for URTI symptoms rather than nasal normal saline (30%), mefenamic acid (11%) and paracetamol (33%). We asked parents if they believed that antibiotics were helpful in treating a variety of symptoms. Although very few parents would ask for antibiotics for URTI symptoms (10%), cough (13%) or vomiting (11%), many would expect their pediatrician to prescribe an antibiotic if their child had earache (51%), fever (41%), or sore throat (27%). Interestingly, parents denied that they would purchase antibiotics over the counter without consulting the pediatrician but 6% admitted they would do so if their doctor had previously prescribed an antibiotic for similar symptoms.

The majority of parents (81%) concurred that antibiotics are excessively used without appropriate indications and declared that they would not change pediatrician if he/she didn’t prescribe easily antibiotics (95.5%), while they affirmed they would change their doctor because she/he prescribed easily antibiotics (66.5%).

According to 88% of parents, their concern regarding secondary complications of upper respiratory tract infections would lead them to seek medical advice, while 60% agree that it is best to avoid the use of antibiotics for uncomplicated URTIs. Finally, the vast majority of parents (98%) agree that education of both parents and pediatricians on the judicious use of antibiotics is necessary.

#### 3.2.3. Section C (Practices)

Few parents (9%) admitted that they were left discontent from their pediatrician whenever he/she didn’t prescribe antibiotics. However, only 59% would query their pediatrician whether it was really necessary in case he/she would give them a prescription or would praise their doctor when he/she elected not to administer antibiotics. In another question, the majority of parents (90%) stated that the side effects of antimicrobial use are important. Very few parents admitted that they would request antibiotics openly (8.5%) or when diagnosis is not certain (6.5%) or over the phone without a preceding office visit (9%), while very few (4.5%) believe that their pediatrician prescribed antibiotics just because parents asked for them. A significant proportion of parents (90%) believe they have been well informed on the judicious use of antibiotics, admit their doctor has spent time explaining the child’s disease and whether he should or not administer antibiotics and more importantly, 97% stated they precisely follow pediatricians’ instructions.

### 3.3. Demographic Factors Associated with Parental KAP

Residence area, ethnicity, parental age, sex and educational level were identified as significant factors associated with knowledge, attitudes and practices of parents concerning judicious antibiotic use for URTI’s. Table 3 depicts most identified associations. Furthermore, parents identifying their doctor as their friend/relative (rather than having a strictly professional relationship with him/her) were more satisfied in reference to the amount of information provided concerning antibiotic use (RR = 1.06, 95%CI 1.03–1.10) and were more likely to praise their pediatrician for withholding antibiotic administration (RR = 1.21, 95%CI 1.11–1.33). Parents with a single child were more likely to feel they have inadequate information considering the judicious use of antibiotics (RR = 0.92, 95%CI 0.86–0.99) and admit they worry more than other parents (RR = 1.33, 95%CI 1.12–1.59). Finally, parents that have good access to the health system consider themselves to be less anxious about their child’s health (RR = 0.75, 95%CI 0.59–0.97).

### 3.4. Logistic Regression Analysis

Possible association of demographic factors with antibiotic misuse was further assessed by applying logistic regression analysis. According to the answers given to the six groups of questions formed for this purpose (Table 1), parents were divided into those with and without antibiotic misuse. Association of demographic factors with knowledge, attitudes and practices concerning antibiotic misuse in children with URI’s are shown in Table 4. Parents’ educational level remained into the model in all six groups of questions.

### 3.5. Pediatricians’ View

Although most pediatricians (>97%) agreed that URTIs are self limited and antibiotic misuse increases antibiotic resistance, 60% agreed that most pediatricians would administer antibiotics for URTIs under parental pressure. Furthermore, although most pediatricians denied they would ever prescribe antibiotics to please the parents, to avoid parents seeking medical help from another pediatrician or because parents insisted, almost 25% would prescribe antibiotics whenever fever persisted for more than 3 days and 15% would do so if the family was leaving on vacation. While all denied administration of antibiotics for common cold or runny nose, 18% and 49% would prescribe antibiotics for croup and otitis with effusion, respectively. While less than 40% reported they believe that parents would appreciate their withholding antibiotics for URTI, more than two thirds reported that they feel that parents want them to administer antibiotics even when their use is not medically necessary. Finally more than 60% reported that parents regularly ask for antibiotics for URTIs.

Comparing the answers given by the pediatricians from Larnaca and Limassol it is worth noticing that pediatricians practicing in Larnaca were more likely to administer antibiotics in cases were fever lasted for more than 3 days (RR = 6.59; 95%CI 0.91–47.76, p = 0.039) and believed that parents expect them to prescribe antibiotics for URTI’s (RR = 2.51; 95%CI 1.32–4.78, p = 0.001) while they were less likely to be praised by parents when withholding antibiotics (RR = 0.28; 95%CI 0.09–0.84, p = 0.008).

## 4. Discussion

This is the first study on KAP from parents in Cyprus concerning antibiotic use in young children with URTI’s. This is a cross-sectional study that included a large number of parents sampled by a stratified geographic clustering methodology. The overall response rate was 69.3%. Although this is lower than that reported in other KAP studies where face-to-face or telephone interviews are used, our methodology using self-answered questionnaires increases the precision of information obtained [15,16]. This methodology was preferred since direct parental interviewing might both involve interviewees’ influence as well as interviewers’ bias in the interpretation of parental answer. Moreover, the probability of the responders’ embarrassment towards the interviewer would affect the quality of their answers [17]. Additionally, in order to interview a large cohort of parents, a large number of interviewers would have to be trained to be sent to interview the parents, which was impractical. Finally, the variability among the interviewers could not be excluded. Using the self-answered methodology however, each responder received the same set of questions phrased in exactly the same way, so the answers were derived in a more objective way. Distribution of the questionnaires by teachers and including a letter explaining the importance of the study probably increased parents’ compliance. This study indicates that school based sampling is helpful and achieves high response rates when compared to randomly sending questionnaires to parents by mail [18].

The response rate was significantly higher in Larnaca compared to Limassol (p ≤ 0.001). Differences in response rates between different geographic areas have been reported and may be explained by differences among the demographic characteristics of parents [14]. Parents from Larnaca had a higher median age and were less likely to be immigrants (Table 2). However, in contrast to previous published studies, parents from Limassol, the area with lower response rate, had a higher education (although not at a significant different level) and had a higher percentage of urban residence when compared to parents from Larnaca [14]. Other factors including idiosyncrasy and perception of importance of health issues may indeed influence response rates in such studies [19]. Interestingly, pediatricians practicing in Larnaca seemed to be more “pressured” by parents to prescribe antibiotics when compared to those from Limassol, suggesting that people residing in Larnaca are more concerned about febrile URTIs.

Six couples of repetitive or contradictory questions were included in order to exclude questionnaires filled in randomly or incoherently. Overall 1.7% questionnaires were not further analyzed. This is a similar percentage as compared to previous studies [14]. As expected, low parental educational level was strongly associated with exclusion of questionnaire due to inconsistency.

In Cyprus, as in Greece and other Mediterranean countries, children’s primary care is provided by private pediatricians. In Cyprus, most have been trained in Greece and Great Britain. Overall parents appeared to have a trusting relationship with their private pediatrician, most (97.5%) declared they follow their exact recommendations, two thirds of parents were happy with their access to the healthcare system and more than a third declared that they had an intimate relationship with their child’s pediatrician. Most parents identified their pediatrician as the major source of information regarding use and misuse of antibiotics whereas in other KAP studies television has been identified as the preferred source of information [20]. Although parents were overall knowledgeable in regard to antibiotic administration during children’s URTIs and their side effects, it is worth noticing that most parents believe that scientists continuously discover new antimicrobials and therefore do not realize the problems faced by the scientific society with shortage of new antimicrobials in the production pipeline [5]. Furthermore, although they are able to recognize that URTIs are usually viral and that fever should not be treated with antibiotics, many believe that antibiotics decrease duration or complications.

Most parents’ expectation for antibiotic prescription is high, therefore, further parental education is needed concerning when to visit a doctor during uncomplicated URTIs, as well as on the recent guidelines for the symptomatic treatment of otitis media in children over 2 years old [21].

In a recent Greek study, it was shown that a significant proportion of antibiotic outpatient consumption is secondary to over the counter purchase, while in another study involving pharmacists it was depicted that over the counter purchase of antibiotics is easy [22,23]. In contrast, in a study from the University of Thessaly Greece, referring to parents alone, it was revealed that although parents have the opportunity to purchase antibiotics over the counter, less than 10% of parents would consider doing so [24]. In our study, very few parents admitted administering antibiotics to their children suffering from URTIs without first consulting their pediatrician, indicating that parents follow their doctors advice. This is in accordance with a recent study indicating that Cyprus has the lowest over the counter consumption among several other South Mediterranean countries [25].

Clearly demographic factors such as residence area, ethnicity, parental age, sex and educational level are associated with knowledge, attitudes and practices concerning judicious use of antibiotics (Table 3). Older parental age and higher educational level were clearly associated with better knowledge (Table 3). While younger parents were worried about fever, older parents were more likely to worry by the altered behavior of their child during a URTI, acknowledge that antibiotics are overused, that it is better to watch a child with URTI rather than administer antibiotics and declared concern over the side effects of antibiotic treatment. Immigrant parents declared they would be unsatisfied if antibiotics were not prescribed and would change pediatrician if he/she did not prescribe antibiotics easily. When logistic regression was applied the most important factor associated with correct parental knowledge, attitudes and practices on judicious use of antibiotics during childhood URTI’s was parental educational level (Table 4). Ethnicity was also related to knowledge concerning antibiotic use while immigrants were more likely to purchase antibiotics over the counter. Parental demographics such as age, educational level, ethnicity and income have been previously been identified by others as important factors explaining KAPs related to antibiotic consumption [9–11,26–28].

Overall, it appears that the over consumption of antibiotics in Cyprus is not overtly driven by parental pressure. Moreover, parents identifying their pediatrician as a relative/friend are content with received education and more likely to even praise them for not prescribing antibiotics. These findings are supported by others, since parents’ satisfaction is shown to be related to the time spent with the doctor rather than the recommended treatment [9]. Therefore, it appears that pediatricians in Cyprus have made a significant effort over the years to build a trusted relationship and educate parents on the use of antibiotics in children with URTI’s. These findings are in accordance with recent data from the Armed project that suggest that in Cyprus self-medication rates are low while the prevalence of *E. coli* and *Streptococcus pneumoniae* resistance is low when compared to other countries in the Euro-Mediterranean area [23,29,30]. However, increased prevalence of methicillin-resistant *Staphylococcus aureus* (MRSA) in invasive isolates has been recently reported [31]. Moreover, outpatient antibiotic consumption in Cyprus is high when compared to other EU countries [12]. It is possible therefore that as described in other European countries primary care physicians consistently prescribe antibiotics for URTIs despite current existing evidence of little benefit and universal pressure for minimizing antibiotic use [32]. This is supported by our finding that about 40% of parents have received telephone instructions for antibiotic administration as well as the recognition from over a quarter of parents (28%) that they have directly asked their pediatrician to prescribe antibiotic treatment. However, in contrast to parents, pediatricians reported that 60% of parents directly ask for antibiotics. So, although the parents’ view supports that antibiotic prescription is not parental driven, the small sub-study on pediatricians view showed that pediatricians (66%) feel that indeed parents expect them to administer antibiotics.

## 5. Limitations

The main limitation of this study is that the data provided is of local interest. However, the conclusions could also refer to other countries where private pediatric service predominates or other countries with similar culture such as Greece or other countries in the Mediterranean area. The response rate of <70% is considered low when compared to other studies using different methodology [15,16]. On the other hand, using self-answered questionnaires possibly increases the proportion of honesty in the given answers. A major limitation of the study is the small number of pediatricians interviewed. However, statistical significant results depict the existing gap between parents and pediatricians and may clarify the ways to go further towards the reduction of antibiotic consumption.

## 6. Conclusions

This study indicates that in Cyprus, parents are satisfied by the care provided and recognize their pediatricians as their main source of information. Although, it appears that antibiotic misuse is not driven by parental pressure, pediatricians’ view implies that medical doctors feel that the majority of parents wish for antibiotic administration for their children’s URTIs. It is therefore concluded that simultaneous well structured interventions towards both pediatricians and parents are needed to improve antibiotic consumption.

## Figures and Tables

**Table 1.** Questions selected for the logistic regression analysis by section.

**Section A (knowledge)**	**G1**	Q16) Parents who have recognized none or only 1 out of 4 common commercial names of antibiotics
	**G2**	Q17) Antibiotic should be administered in any case, once a child has fever.
		Q18) As most of the Upper Respiratory Tract Infections (like cold, flu, sore throat, ear infection) are of viral cause, they should not be treated with antibiotics.
		Q19) If a child suffers from flu or cold, resolution of symptoms will be quicker if antibiotic is given on time.
		Q22) When antibiotics are administered for no special reason, their efficacy is decreased and bacteria become more resistant.

**Section B (attitudes)**	**G3**	Q27) How often would you like your pediatrician to perscribe antibiotics for your child when it suffers from cold, ear pain, sore throat, cough, vomit, fever, runny nose?
	**G4**	Q28) How often would you give your child antibiotics without previous pediatricians’ consult?
	**G5**	Q30) Would you change your pediatrician because according to your opinion he/she does not prescribe antibiotics often enough for your child?
		Q31) Would you change your pediatrician because according to your opinion he/she prescribes antibiotics for your child very often?
		Q32) Would you reuse an antibiotic which you had used in the past if your child presents similar symptoms?
		Q34) Do you think that most of the Upper Respiratory Infections will be self—cured even without the use of antibiotics?

**Section C (practices)**	**G6**	Q42) Would you be unhappy if your pediatrician does not prescribe an antibiotic for your child’s Upper Respiratory Infection?
		Q45) How often does your pediatrician recommend antibiotic therapy over the phone?
		Q46) In case you strongly wish your child to receive antibiotics, how often do you directly request it?

**Table 2.** Demogarphic characteristics of parents.

**Demographic characteristics**	**Larnaca (N = 778) n/Total (%)**	**Limassol (N = 716) n/Total (%)**	***p*-value**
Mother responding (%)	614/778 (78.9)	541/701 (77.2)	0.418
Median age (years) (IQR)	36 (33–40)	35 (32–39)	**<0.001**
Having insurance (%)	463/778 (59.5)	430/695 (61.9)	0.355
Having private insurance *	379/463 (81.9)	349/422 (82.7)	0.743
College/University maternal education (%)	285/772 (36.9)	291/696 (41.8)	0.055
College/University paternal education (%)	263/756 (34.8)	250/668 (37.4)	0.301
High monthly income (%) ^#^	667/778 (85,7)	587/686 (85,6)	0.929
Foreign born (%)	27/778 (3.5)	50/685 (7.3)	**0.001**
Urban residence (%)	531/778 (68.3)	634/708 (89.5)	**<0.001**
One child (%)	71/774 (9.2)	101/706 (14.3)	**<0.001**
Single parent family (%)	72/777 (9.3)	55/715 (7.7)	0.276
Child with recurrent URI’s (%)	91/778 (11.7)	75/688 (10.9)	0.631
Good access to healthcare system (%) ^#^	733/767 (95,6)	619/652 (94,9)	0.578
Consider pediatrician relative/friend (%) ^#^	298/775 (38.5)	231/653 (35.4)	0.230

* % among those with insurance.

# self perception of status.

**Table 3.** Demographic factors associated with parental KAP.

**Factor**	**Question**	RR* (95%CI)	***P*-value**
**Residence area (Larnaca *versus* Limassol)**	Q15_7. I have not had any information concerning judicious antibiotic use	2.17 (1.15–4.12)	0.014
	Q30. Would you change pediatrician because he administers antibiotics too often?	0.59 (0.36–0.97)	0.036
	Q46. How often would you directly ask from your pediatrician to prescribe antibiotics?	1.61 (1.12–2.30)	0.008
	Q50. How often do you believe that your pediatrician administered antibiotics because you requested it?	1.74 (1.05–2.89)	0.028

**Parental age (>35years *versus* ≤35 years) Parental sex (father *versus* mother)**	Q15_2. I have had been informed by TV concerning judicious antibiotic use	1.49 (1.16–1.92)	0.002
	Q15_4. I have had been informed by the newspapers concerning judicious antibiotic use	1.78 (1.31–2.42)	<0.001
	Q18. Most URTI’s are self limited	1.16 (1.05–1.28)	0.004
	Q26_7. Altered behavior would lead me at the pediatricians office for advise	1.25 (1.08–1.46)	0.003
	Q34. Do you believe it is better to follow a child with URTI rather than prescribing antibiotics?	1.20 (1.08–1.34)	0.001
	Q35. Would you be more prone to ask for antibiotics if your child had often URI’s	0.72 (0.57–0.92)	0.008
	Q39. How much information on the judicious antibiotic use have you had?	1.04 (1.01–1.08)	0.019
	Q41. Are you concerned about antibiotic side effects?	1.05 (1.01–1.08)	0.010
	Q15_2. I have had been informed by TV concerning judicious antibiotic use.	0.72 (0.52–0.99)	0.042
	Q15_7. I have not had any information concerning judicious antibiotic use	2.29 (1.26–4.16)	0.006
	Q19. Antibiotics shorten URTI duration	1.24 (1.09–1.40)	0.002
	Q21. Antibiotics do not have side effects	1.61 (1.02–2.53)	0.040
	Q27_1. How often would you want your pediatrician prescribe antibiotics for a common cold.	1.81 (1.30–2.52)	0.001
	Q27_4. How often would you want your pediatrician prescribe antibiotics for cough.	1.56 (1.16–2.10)	0.004
	Q27_5. How often would you want your pediatrician prescribe antibiotics for vomiting.	2.06 (1.51–2.82)	<0.001
	Q32. Would you ever use left over antibiotic for a new episode of URI or sore throat?	1.96 (1.01–3.81)	0.043
	Q42. Do you agree you would be left discontent if your pediatrician did not prescribe antibiotics?	0.63 (0.40–0.99)	0.045

**Parental educational level (At least one parent with college education*versus*parents with up to secondary school)**	Q15_4. I have had been informed by the newspapers concerning judicious antibiotic use	0.50 (0.37–0.67)	<0.001
	Q17. When your child has fever, does he always needs antibiotics?	2.31 (1.67–3.19)	<0.001
	Q18. Most URTI’s are self limited	0.75 (0.68–0.82)	<0.001
	Q19. Antibiotics shorten URTI duration	1.73 (1.52–1.97)	<0.001
	Q21. Antibiotics have no side effects	2.23 (1.42–3.51)	<0.001
	Q22. Antibiotic misuse increases antibiotic resistance.	0.92 (0.88–0.95)	<0.001
	Q26_3. Nasal congestion would lead me at the pediatricians office for advise.	1.79 (1.27–2.53)	0.001
	Q27_2. How often would you want your pediatrician to prescribe antibiotics for nasal congestion?	3.90 (2.04–7.45)	<0.001
	Q27_4. How often would you want your pediatrician to prescribe antibiotics for cough?	2.33 (1.70–3.18)	<0.001
	Q28_1. How often would you administer antibiotics to your child over the counter because of reduced time or to avoid visit expenses?	3.17 (1.38–7.28)	0.004
	Q28_2. How often would you administer antibiotics to your child over the counter because you considered disease mild?	3.26 (1.74–6.12)	<0.001
	Q28_3. How often would you administer antibiotics to your child over the counter because he had previously prescribed one for similar symptoms?	1.88 (1.20–2.94)	0.005
	Q34. Do you believe it is better to follow a child with URTI rather than prescribing antibiotics?	0.68 (0.61–0.76)	<0.001
	Q42. Do you agree you would be left discontent when your pediatrician did not prescribe antibiotics?	1.47 (1.05–2.07)	0.024
	Q45. How often do you get instructions concerning antibiotic administration over the phone?	1.46 (1.04–2.04)	0.027

**Parental Ethnicity (Immigrant*versus*Cypriotic Ethnicity)**	Q15_6. I have had been informed concerning judicious antibiotic use by relative	2.86 (1.25–6.54)	0.024
	Q26_2. Fever would lead me at the pediatricians office for advise	0.72 (0.59–0.87)	<0.001
	Q26_4. Ear ache would lead me at the pediatricians office for advise	0.82 (0.71–0.96)	0.001
	Q28_2. How often would you administer antibiotics to your child over the counter because you considered disease mild?	3.61 (1.84–7.07)	0.001
	Q30. Would you change pediatrician because he does not administer antibiotics easily?	3.51 (1.81–6.83)	0.002
	Q32. Would you ever use left over antibiotic for a new episode of URI or sore throat?	4.38 (2.01–9.56)	0.002
	Q42. Do you agree you would be left discontent when your pediatrician did not prescribe antibiotics?	2.63 (1.65–4.19)	<0.001

* RR = refers to what is the relative risk of parents answering correctly to each question according to their demographic factor examined.

**Table 4.** Logistic regression analysis on factors associated with misuse of antibiotics.

**Questions**	**Demographic factor**	**OR**	**95% CI**	***p*-value**
**Group 1**	Parents’ educational level *	1.58	1.11–2.26	0.011
	Having Insurance (No/Yes)	1.49	1.04–2.13	0.031
	Immigrant (Yes/No)	2.21	1.19–4.10	0.012
	Sex (Father/Mother)	2.24	1.54–3.26	<0.001
	Income (high/low)	0.52	0.34–0.79	0.002
	No of children (1 *versus* ≥2)	3.19	2.11–4.83	<0.001
	Frequent URI’s (No/Yes)	2.23	1.16–4.29	0.016

**Group 2**	Parents’ educational level *	7.44	0.93–59.84	0.059
	Immigrant (Yes/No)	6.00	1.21–29.77	0.028

**Group 3**	Parents’ educational level *	2.45	1.08–5.60	0.033
	Sex (Father/Mother)	3.57	1.70–7.50	0.001

**Group 4**	Parents’ educational level *	2.19	1.45–3.32	<0.001
	Immigrant (Yes/No)	2.22	1.09–4.51	0.028

**Group 5**	Parental Age	0.69	0.50–0.95	0.021
	(>35year *versus* ≤35year)	2.88	2.02–4.12	<0.001
	Parents’ educational level *			
	Income (high/low)	0.59	0.40–0.87	0.008

**Group 6**	Parents’ educational level *	2.47	1.38–4.44	0.002
	Residence (urban/rural )	0.56	0.32–0.95	0.032

* Parents’ educational level = Up to secondary school *versus* at least one parent College/University.
